# Fast local fragment chaining using sum-of-pair gap costs

**DOI:** 10.1186/1748-7188-6-4

**Published:** 2011-03-18

**Authors:** Christian Otto, Steve Hoffmann, Jan Gorodkin, Peter F Stadler

**Affiliations:** 1 Bioinformatics Group, Dept. of Computer Science, University of Leipzig, Germany; 2 LIFE - Leipzig Research Center for Civilization Diseases, Universität Leipzig, Germany; 3 Center for non-coding RNAs in Technology and Health (RTH), University of Copenhagen, Denmark; 4 RNomics Group, Fraunhofer Institute for Cell Therapy and Immunology, Leipzig, Germany; 5 Santa Fe Institute, Santa Fe, New Mexico, USA; 6 Department of Theoretical Chemistry, University of Vienna, Austria; 7 Max-Planck-Institute for Mathematics in Sciences, Leipzig, Germany

## Abstract

**Background:**

Fast seed-based alignment heuristics such as BLAST and  BLAT  have become indispensable tools in comparative genomics for all studies aiming at the evolutionary relations of proteins, genes, and non-coding RNAs. This is true in particular for the large mammalian genomes. The sensitivity and specificity of these tools, however, crucially depend on parameters such as seed sizes or maximum expectation values. In settings that require high sensitivity the amount of short local match fragments easily becomes intractable. Then, fragment chaining is a powerful leverage to quickly connect, score, and rank the fragments to improve the specificity.

**Results:**

Here we present a fast and flexible fragment chainer that for the first time also supports a sum-of-pair gap cost model. This model has proven to achieve a higher accuracy and sensitivity in its own field of application. Due to a highly time-efficient index structure our method outperforms the only existing tool for fragment chaining under the linear gap cost model. It can easily be applied to the output generated by alignment tools such as segemehl or BLAST. As an example we consider homology-based searches for human and mouse snoRNAs demonstrating that a highly sensitive BLAST search with subsequent chaining is an attractive option. The sum-of-pair gap costs provide a substantial advantage is this context.

**Conclusions:**

Chaining of short match fragments helps to quickly and accurately identify regions of homology that may not be found using local alignment heuristics alone. By providing both the linear and the sum-of-pair gap cost model, a wider range of application can be covered. The software clasp is available at http://www.bioinf.uni-leipzig.de/Software/clasp/.

## Background

The detection of (potentially) homologous sequence fragments is a basic task in computational biology that underlies all comparative approaches from molecular phylogenetics to gene finding, from detailed analysis of evolutionary patterns of individual genes to global comparisons of genome structure. On genome-wide scales, BLAST[1] has become the bioinformatician's work horse for homology search, with a sensitivity and specificity that is sufficient for most applications in comparative genomics. It is in particular the basis for the currently available genome-wide alignments, which in turn underlie a wide variety of subsequent analyses.

Some specialized tasks such as the search for distant homologs of short structured RNAs [2], require more sensitive techniques. In particular, sequence families exhibiting only short conserved blocks interspersed with highly variable regions are difficult for BLAST or  BLAT[3] because the seeds have to be very short in this case. This typically leads to a huge number of short match fragments that require sophisticated post-processing to discriminate single random hits from sets of adjacent hits potentially indicating true homologs.

The objective of fragment chaining is to efficiently find sets of consistent fragments with a maximal score [4]. The order of fragments is assumed to be congruent in both query and database sequences. While the case of overlapping fragments is explicitly excluded, gaps between fragments are allowed and may be penalized according to different scoring models. In the case of a local fragment chaining, the score of any fragment within a chain must not be smaller than the penalty that is assigned to the gap to the successive fragment. Thus, a chain is a sequence of non-overlapping, i.e., disjoint, ordered fragments and its score is the sum of their fragment scores minus the penalties for any gaps between them. Introduced in sequence alignments [5], fragment chaining may be used in several comparative tasks such as whole genome comparison, cDNA/EST mapping, or identifying regions with conserved synteny as described in [6].

**Figure 1:**
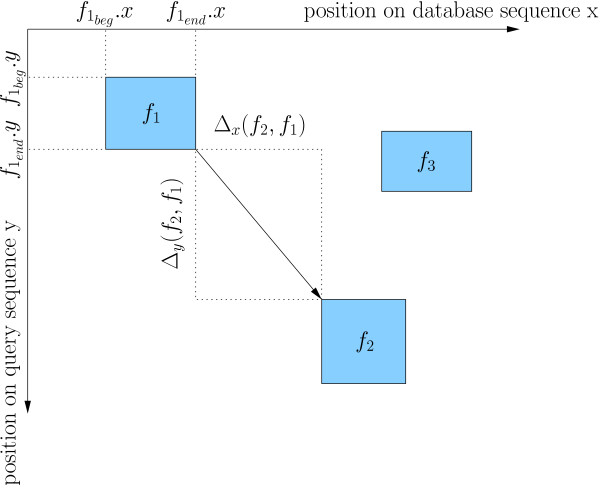
**Graphical representation of fragments and chaining connections**. Graphical representation of fragments as blocks with their respective database and query positions. All valid chaining connections are depicted as edges including their distance on database *x *and query sequence *y*. Note that *f*_1 _and *f*_3 _can not be chained due to their overlap on the query sequence *y*.

Let *f_beg_*.*x*, *f_end_*.*x *denote the start and end position of a fragment *f *in the database sequence *x*. The start and end positions in the query *y *are denoted by *f_beg_*.*y *and *f_end_*.*y*, respectively. Let *f *and *f' *be two non-overlapping ordered fragments, i.e., assume  and . Linear gap costs *g*_1_(*f'*, *f*) between the fragments *f *and *f' *are calculated by:(1)

with , and weighting parameters . Note that the use of weighting parameters in the gap cost model is equivalent to linear weights on fragment scores. A graphical illustration of fragments and chaining connections is shown in Figure 1. For  linear gap costs penalize any distance between fragments on query and database sequence. This scoring system may not be suitable, however, when scattered blocks of local sequence conservation are expected.

The more flexible sum-of-pair gap cost model introduced by Myers and Miller [7] allows to penalize differences of the distances between adjacent fragments on query and database only. The sum-of-pair gap costs *g_sop_*(*f'*, *f*) between non-overlapping ordered fragments *f *and *f' *is given by(2)

with parameters . Intuitively,  expresses the penalty to align an anonymous character with a gap position while  is the penalty to align two anonymous characters. With , the chaining only minimizes the distance difference between fragments.

The software tool CHAINER, a part of CoCoNUT[8,9], implements fragment chaining with linear gap costs. AXTCHAIN, part of the UCSC genome browser pipeline, also uses the linear gap model [10,11]. The tool expects pairwise alignments alignments as input and hence cannot be used "as is" with plain fragment files produced from external applications. The SeqAn library provides algorithms for fragment chaining with different gap cost models [12]. A running tool that implements these models, however, is not available at present.

## Implementation

We implemented the local fragment chaining algorithm, introduced by [4,6]. In addition to the linear gap cost model in CHAINER, the more flexible sum-of-pair gap cost model has been incorporated for the first time in a standalone tool.

The chaining algorithm is based on sparse dynamic programming [13], since for any fragment only a small set of possible predecessors needs to be considered in order to find the optimal one. More precisely, the optimal predecessor is a non-overlapping chain preceding the fragment in both database and query sequence that leads to the maximal combined score considering the gap cost penalty between them. In the case of local fragment chaining, the fragment is chained to the optimal predecessor only if its score is equal to or higher than the necessary gap costs. Using theoretical results on both gap cost models [4], priorities can be assigned to chains in such a way that the optimal predecessor has the maximal priority. Using the line-sweep paradigm, the algorithm scans through the list of fragment start and end points ordered by their database position. For any start point, the optimal predecessor is identified by means of range maximum queries (RMQs) over the set of active chains, i.e., chains only comprised of fragments with already processed end points. The RMQ reports the element with maximal priority within a given range that involves only non-overlapping chains preceding the current fragment in both database and query sequence. For any end point, a novel chain is generated by connecting the optimal predecessor to the current fragment and is marked as active. In the end, the algorithm groups together chains with common first fragment and reports the best-scoring chain of each group. Note that a fragment does not necessarily have to be the first fragment of any best-scoring chain.

**Figure 2:**
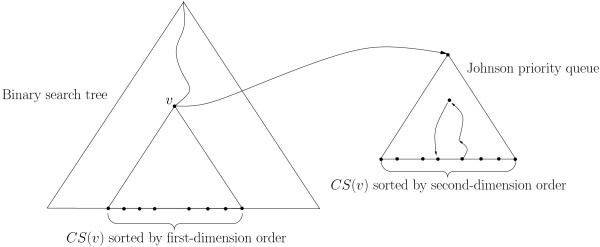
**Illustration of a range tree padded with Johnson priority queues as stratified tree structure**. Illustration of the stratified tree structure consisting of a primary binary search tree sorted by the first-dimension order padded with Johnson priority queues in each node sorted by the second-dimension order.

In contrast to CHAINER, we implemented Johnson priority queues [14] and range trees padded with Johnson priority queues instead of simple kd-trees to support RMQs. One-dimensional RMQs are answered using Johnson priority queues, i.e., semi-dynamic tree structures permitting non-recursive binary searches on tree paths. The priority domain, i.e., the range of possible priorities, is defined at the point of initialization. Hence, the balanced tree structure provides binary search information at tree nodes. In order to condense the priority domain, we linked the priorities to the sorting order of all potential elements. Let *n *be the length of the priority domain. Johnson priority queues support predecessor, successor, insert, and delete operations in  time. To efficiently implement sum-of-pair gap costs we need to consider two distinct sorting dimensions [4]. For the two-dimensional RMQs, range trees were padded with Johnson queues (see Figure 2). More precisely, the range tree is a primary binary search tree for all elements sorted by their first-dimension order. Additionally, each node *v *stores a Johnson priority queue containing all elements in the subtree beneath *v*, referred to as the canonical subset *CS*(*v*). Elements in Johnson priority queues are sorted by the second-dimension order. In summary, the implemented fragment chaining algorithm requires  in time with linear gap costs and  in time with sum-of-pair gap costs.

Because the database is typically much larger than the query sequence, we introduced a novel clustering approach to facilitate local fragment chaining. The basic idea is to improve the running time by assigning fragments to clusters that can be chained separately from each other without resulting in different chaining outcome. It first pools neighboring fragments in a single linear scan using the following observation: Let *f *and *f' *be two adjacent non-overlapping fragments on the database sequence. Clearly, *f' *and *f *may never be chained and can be assigned to different clusters if(3)

where *max_score _*is the highest possible chain score and *max_y _*is the maximal distance of fragments on the query sequence. Note that *max_score _*is bounded from above by the length of the query multiplied by the maximal score per fragment position. Estimates of *max_score _*and *max_y _*are calculated and updated during the linear scan. Hence, the clustering is accomplished with only one linear scan consuming only a negligible amount of additional memory. Subsequently, rather than applying the chaining algorithm to the entire list of fragments, each of the clusters can be chained separately, improving both running time and memory consumption. In the worst case, all fragments are in the same cluster leading to the same performance as without clustering. We incorporated clustering in local fragment chaining with linear gap costs using an analogous condition. Note that fragments from different queries or database sequences (e.g., chromosomes) can be processed in a single pass by our tool but are generally chained separately from each other (even without use of clustering).

More details on the implemented data structures, their worst-case time complexities, and the chaining algorithm can be found in the Additional file 1. Note that the algorithm is implemented for two-dimensional fragments only, i.e., fragments with position information on one query and one database sequence, due to its intended area of application.

## Results and Discussion

### Performance Tests

**Figure 3:**
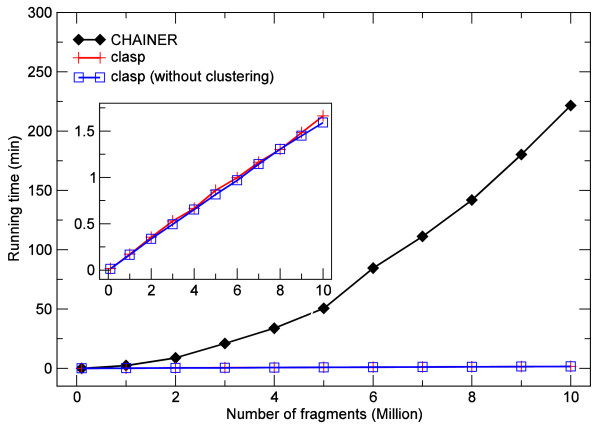
**Comparison of running times between **clasp**and **CHAINER. Average running time for clasp (linear gap costs with , ) and CHAINER (options: -l -lw 1) by chaining different numbers of randomly generated fragments of length 100 between a 1 KB large query sequence from a virtual 100 KB large database under the linear gap cost model. Comparison of running time between use of clustering (by default) and no clustering in clasp with equal data sets shown in inlay plot (same units on axes).

**Figure 4:**
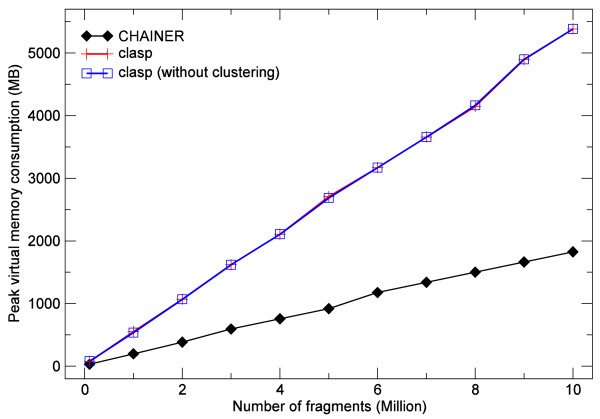
**Comparison of peak virtual memory usage between **clasp**and **CHAINER. Peak virtual memory usage for clasp using linear gap costs with ,  (with and without clustering) and CHAINER (with options -l -lw 1) by chaining different number of randomly generated fragments of length 100 between a 1 KB large query sequence from a virtual 100 KB large database under the linear gap cost model.

In order to evaluate the performance of clasp using linear gap costs with  and , we compared it to CHAINER v3.0 with options -l -lw 1 producing comparable scores. Each simulated data set contained fragments of length 100 covering 1 KB query sequences, uniformly sampled from a virtual 100 KB large database. Scores were sampled from a normal distribution. Both programs were executed single-threaded on the same 64-Bit machine with equal data sets. Moreover, the performance of clasp was analyzed with and without the use of our clustering method. The results for different numbers of sampled fragments are shown in Figure 3 and 4. We measured the performance in terms of running time in user mode and peak virtual memory consumption. If not disabled, the clustering procedure as an integral part of our algorithm is naturally included in all measurements of running time and memory consumption.

In terms of running time, clasp (with and without clustering) outperforms CHAINER in any tested setting at the expense of a three-fold increased memory consumption during execution. Due to the uniform distribution of query sequences the use of clustering only leads to a minor performance improvement. In each test case, the quality of the chains was assessed by comparing the distributions of chain scores reported by both programs. In a few cases, only marginal differences between clasp and CHAINER were observed. These differences do not require further attention from our side.

### Homology searches with Human box H/ACA snoRNAs

To assess the performance of clasp in real-life applications, a sequence-based homology search was carried out. Human box H/ACA snoRNA families, an important class of structured RNAs, were selected to identify potentially homologous regions in entire genome of *Mus musculus*. BLAST fails to report sufficiently long hits but, e.g., in the case of the 134 nt long Human H/ACA snoRNA 42 (SNORA42 in the snoRNABase [15]), dumps more than 10 millions short hits in the mouse genome when executed in a very sensitive mode with small word sizes and high expectation values (options: -W 8 -e 1e+20 -F F).

**Figure 5:**
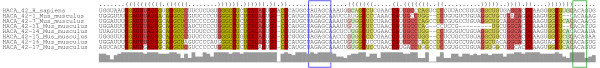
**Alignment of Human H/ACA snoRNA 42 and homologous H/ACA snoRNA candidates in mouse retrieved by **BLAST**and **clasp**with sum-of-pair gap costs**. Alignment of the Human H/ACA snoRNA 42 (SNORA42 in the snoRNABase) and 7 H/ACA snoRNA candidates in mouse retrieved by combined use of BLAST (with options -W 8 -e 1e+20 -F F) and clasp (sum-of-pair gap costs with , , fragment scores according to the length of the BLAST hit, and a minimal required chain score of 30). Sequence alignment and consensus secondary structure were computed using ClustalW and RNAalifold with constraints, i.e. demanding single-stranded regions at the H-box (blue rectangle) and ACA-motif (green rectangle).

We executed clasp using the sum-of-pair cost model with ,  (only punish for distance differences with half of the match score) fragment scores according to the length of the BLAST hit, and a minimal required chain score of 30. The use of clustering greatly reduced the memory requirements: Instead of more than 100 GB, the fragment chaining on the 1.2 GB BLAST output file consumed only 1.6 GB and took less than 5 minutes on a single 2.33 GHz 64-Bit Intel Xeon CPU. In the end, clasp reported 17 chains in disjoint regions of the mouse genome. In order to check for conservation of H-box and the ACA-motif, the mouse candidates were aligned to the initial Human H/ACA snoRNA 42 sequence using the multiple alignment tool ClustalW[16]. We further checked the secondary structure conservation and stability by folding each candidate using RNAsubopt[17] with constraints, i.e., demanding single-stranded regions at the H-box and ACA-motif. In total, we identified 7 of the 17 regions as H/ACA snoRNA candidates homologous to the Human H/ACA snoRNA 42 (see Additional file 2). The sequence alignment of the final candidates and the Human H/ACA snoRNA 42 including consensus secondary structure and sequence conservation is shown in Figure 5. By checking with previous annotations, all of the final candidates were confirmed as snoRNA orthologs by the Ensembl database [18,19]. However, ncRNAs in the Ensembl database were annotated using extensive Infernal screens with Rfam covariance models [20], i.e., profile stochastic context-free grammars comprising primary sequence and secondary structure information.

**Figure 6:**
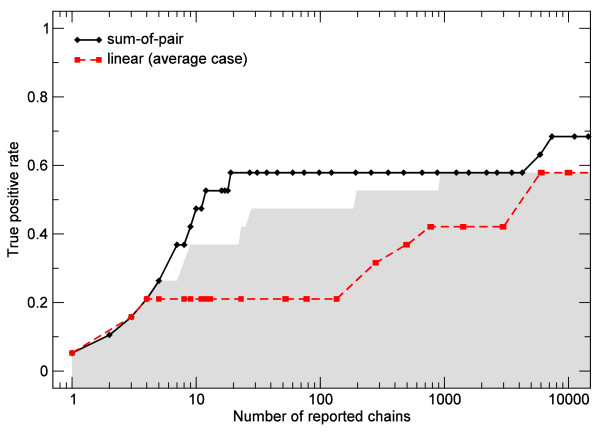
**Comparison between sum-of-pair gap costs and linear gap costs in the retrieval of Ensemble annotated SNORA42 homologs in mouse**. The figure shows the true positive rate (TPR) for identifying Ensembl-annotated Human SNORA42 homologs with respect to the total number of reported chains for both linear and sum-of-pair gap cost models. In case of the linear gap cost model, a wide range of values are selected for the weighting parameters  and , i.e., . In the sum-of-pair gap cost model, the parameters  and  are chosen. Note that the number of reported chains for a given parameter set is entirely determined by the minimal required chain score. The average TPR of clasp using the linear gap cost model (, , dashed red line) is significantly lower compared to sum-of-pair gap cost model (solid black line). However, the performance of chaining with linear gap cost models heavily depends on the selection of parameters (shaded area).

To illustrate the benefits of the sum-of-pair gap cost model, we additionally compared the performance of clasp using both models in a snoRNA homology search experiment. We selected the entire set of 19 annotated Human SNORA42 homologs in the Ensembl database as a positive set. In the comparative study, clasp was executed with sum-of-pair gap costs (with , ) and linear gap costs with several different parameter selections . For each parameter setting, the true positive rate (i.e., the fraction of SNORA42 that was covered by at least one chain) was recorded with respect to the total number of reported chains, a function of the minimal required chain score. In the average as well as the best case of parameter selection the linear gap cost is outperformed by the sum-of-pair model (Figure 6). Using sum-of-pair with  and , 11 out of 19 annotated snoRNAs are among the 19 best chains. With linear gap costs and optimal parameter settings (), a list of 900 best scoring chains has to be scanned to find the same number of annotated snoRNAs (49-fold increase). With suboptimal parameters, about 6000 chains (314-fold increase) need to be screened on average to retrieve the same amount of snoRNAs. Note that alternative weighting functions of fragment scores or in the linear gap cost model, e.g., affine or non-linear functions, are currently not implemented but are subject to further research.

**Figure 7:**
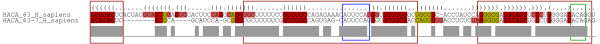
**Alignment of Human H/ACA snoRNA 77 and paralogous H/ACA snoRNA candidate retrieved by **BLAST**and **clasp**with sum-of-pair gap costs**. Alignment of the Human H/ACA snoRNA 77 (SNORA77 in the snoRNABase) and a novel paralogous H/ACA snoRNA candidate retrieved by combined use of BLAST (options: -W 8 -e 1e+20 -F F) and clasp (sum-of-pair gap costs with , , fragment scores according to the length of the BLAST hit, and a minimal required chain score of 30). It shows a highly conserved H-box (blue rectangle) and ACA-motif (green rectangle) as well as high secondary structure conservation with two separate stem loop regions. Despite a sequence identity score of 70 reported by ClustalW, BLAST was capable to retrieve only 3 short regions, marked by red rectangles, none of which individually provides sufficient evidence of homology.

Using the same methods and parameters as in the search for homologs, the Human genome was screened with the entire set of annotated Human H/ACA snoRNAs in the snoRNABase (107 sequences with a median length of 134 nt) to identify divergent paralogs. Fragment chaining of the 155 GB of BLAST output, comprising more than 1.3 × 10^9 ^hits, took only 11 hours on a single 2.27 GHz 64-Bit Intel Xeon CPU with a peak virtual memory consumption of 18 GB. In the end, 2294 non-overlapping chains were reported with sum-of-pair gap costs. Requiring conservation in the H-box, the ACA-motif, as well as in the secondary structure, 1550 candidates were retained. To filter out non-paralogous regions different sequence identity cutoffs in the ClustalW alignment to known Human H/ACA snoRNAs were applied. The number of remaining chains including their fragment counts and their overlap with existing annotations are summarized in Table 1. The annotations comprise the snoRNABase, the set of snoRNAs and snoRNA pseudogenes from the Ensembl database and the Eddy-BLAST-snorna lib. The latter one is a set of snoRNA candidates retrieved by post-processing WU-BLAST screens starting from Human snoRNAs [21]. By requiring more than 70% sequence identity to a snoRNABase annotated sequence, our set of final candidates comprises 295 sequence of which 187 are not annotated in the snoRNABase (see Additional file 3). 29 final candidates were not previously annotated in the snoRNABase and only detectable by chaining two or more BLAST hits. Overall, more than 98% of the final candidates have been annotated previously, most of them by the covariance approach of the Ensembl database. This points out the high accuracy of this rather simple homology search. Figure 7 shows a region that was identified with a chain of only 3 fragments. It is a paralog to the Human H/ACA snoRNA 77 (SNORA77 in the snoRNABase) from the set of remaining unknown snoRNA candidates.

**Table 1:** Novel candidates of Human H/ACA snoRNA paralogs

			annotated candidate regions in %			
sequence Identity	fragments per chain	number of chains	snoRNABase	Ensembl	Eddy-BLAST-snornalib	unknown
> 60%	1	286	37.8	94.4	84.3	6
	2	29	0	69	86.2	3
	≥ 3	10	0	70	60	3
	all	325	33.2	91.4	83.7	12

> 70%	1	266	40.6	97.7	84.6	3
	2	21	0	85.7	95.2	0
	≥ 3	8	0	87.5	75	1
	all	295	36.6	96.6	85.1	4

> 80%	1	233	46.4	98.7	85	1
	2	10	0	90	100	0
	≥ 3	2	0	100	100	0
	all	245	44.1	98.4	85.7	1

Summary of H/ACA snoRNA candidates in *Homo sapiens *including their fragment counts and their overlap with previous annotations, i.e., the snoRNABase, the set of snoRNAs and snoRNA pseudogenes from the Ensembl database and the Eddy-BLAST-snornalib in the UCSC RNAGenes track.

The candidates were retrieved by combined use of BLAST (with options -W 8 -e 1e+20 -F F) and clasp (sum-of-pair gap costs with , , fragment scores according to the length of the BLAST hit, and a minimal required chain score of 30) with the entire set of Human H/ACA snoRNAs, annotated in the snoRNABase. Each candidate shows a highly conserved H box and ACA motif as well as high secondary structure conservation with two separate stem loop regions. Moreover, several different sequence identity scores in the ClustalW alignment to a known Human H/ACA snoRNA were required.

## Conclusions

Commonly used local alignment heuristics may fail to retrieve sequence families with scattered conservation. Chaining of short match fragments can overcome this limitation, thereby substantially enhancing the effective sensitivity of BLAST and similar approaches in homology search. The clasp tool implements a fast local fragment chaining algorithm supporting the linear and the sum-of-pair gap model. The latter is available for the first time in a running tool and is particularly suitable to cope with scattered sequence conservation, e.g., evolutionary conserved structured ncRNAs. In this field of application, it outperforms optimized linear gap models in terms of accuracy and sensitivity. We showed that the usage of Johnson priority queues greatly improves the runtime performance in comparison to the only existing fragment chaining tool CHAINER. The presented clustering approach facilitates clasp to tackle large amounts of short match data by alignment heuristics such as segemehl or BLAST. In a simple homology search with H/ACA snoRNAs, we were able to identify 7 H/ACA snoRNA candidates in mouse, all confirmed by the annotation in the Ensembl database. A large-scale survey for Human H/ACA snoRNA paralogs yielded 295 candidates with more than 70% sequence identity to Human H/ACA snoRNAs from the snoRNABase. More than 98% of the candidates have been annotated previously, in particular with respect to the extensive Ensembl ncRNA screens, emphasizing the high specificity of this rather simple homology search.

## Availability and requirements

Project name: clasp

Project home page: http://www.bioinf.uni-leipzig.de/Software/clasp/

Operating system(s): platform independent

Programming language: C

Other requirements: none

License: GNU GPL

Any restrictions to use by non-academics: Note that a license is needed to include the source code from the clasp in commercial software projects.

## Competing interests

The authors declare that they have no competing interests.

## Authors' contributions

CO implemented the software and drafted the manuscript. SH implemented parts of the tool and contributed to the manuscript. JG and PFS initiated and designed the project and contributed to the manuscript. All authors read and approved the final manuscript.

## Supplementary Material

Additional file 1 **More detailed description of data structures and chaining algorithm**. Text file containing a more detailed description on the implemented data structures, i.e., Johnson priority queues and range trees, as well as on the chaining algorithm with both gap costs models and the clustering approach. Click here for file

Additional file 2 **Candidates of Human H/ACA snoRNA 42 homologs in mouse**. Archive file containing genomic coordinates and sequences of the 7 final candidates of Human H/ACA snoRNA 42 (SNORA42) homologs found in mouse (mm9). Click here for file

Additional file 3 **Candidates of Human H/ACA snoRNA paralogs**. Archive file containing genomic coordinates and sequences of the final candidates of Human H/ACA snoRNAs paralogs, i.e., candidate set requiring more than 70% sequence identity to a snoRNABase annotated sequence, found in human (hg18) including the query sequences from the snoRNABase. Click here for file
