# Supplementary material for: Fast local fragment chaining using sum-of-pair gap costs
Source: Algorithms Mol Biol. 2011 Mar 18;6:4. doi: 10.1186/1748-7188-6-4 (PMC3072320; doi:10.1186/1748-7188-6-4)
Supplement: Additional file 1 — More detailed description of data structures and chaining algorithm. Text file containing a more detailed description on the implemented data structures, i.e., Johnson priority queues and range trees, as well as on the chaining algorithm with both gap costs models and the clustering approach. [file 1748-7188-6-4-S1.PDF]

More detailed description of the data structures and the chaining algorithm

This document covers additional information on the implemented data structures and a more detailed description of the chaining algorithm itself.

1 Johnson priority queues

As an extension of the concept of van Emde Boas trees [1], Johnson developed a non-recursive approach that permits binary searches on tree paths in order to perform efficient insert, delete, predecessor, and successor operations [2]. The search for the successor of a leaf in a search tree is composed of two steps. In the first one, the first internal node on the path from the leaf to the root is located that does have a right child node. The nodes on this path are denoted as right base with the leaf as right base bottom and the node with non-empty right child as right base top. Left base bottom and left base top are defined in similar manner. During the second part, the predecessor is identified as the left-most leaf in the subtree rooted by the right child of that right base top. While the path in the first part is unique there are multiple possible paths during the second part depending on the leaves initialized in the subtree. However, the second step is simply the jump from the right child node to its left base bottom. If this base information is stored in each node, searches for predecessors or successors will only require one bottom-up binary search (until an internal node with the required right (or left) base information is found) and two jump, one upwards and one downwards, using the base information. Due to the semi-dynamic tree structure, the number of elements (leaves) and hence the height h of the tree is defined at the point of initialization and base information can be stored in internal nodes where binary searches from any leaf to the root may jump, i.e., all nodes at depths $h-1$, $h-2$, $h-4$, ..., 1. In such a way, predecessor and successor operations are supported in $\mathcal{O}(\log(\log(n)))$ in time. Note that predecessor or successor queries of existing leaves can be answered in $\mathcal{O}(1)$. Hence, range queries require at most one binary search for the predecessor of the given upper boundary while the other elements within the range are identified in constant time. In summary, Johnson priority queues support one-dimensional range queries with a worst-time complexity of $\mathcal{O}(\log(\log(n)) + z)$ where z elements are within the given range. Certainly, the base information needs to be updated during each insert or delete operation that requires $\mathcal{O}(\log(\log(n)))$ in time using

binary searches as well. In summary, Johnson presented a semi-dynamic priority queue that supports range queries very efficiently by use of predecessor and successor operations.

2 Range trees

In the previous section, Johnson priority queues as one-dimensional sorted lists have been introduced to permit several operations, e.g., predecessor or successor searches. In such a way, it is possible to traverse a priority queue according to its sorting order from minimum to maximum or vice versa. Furthermore, requests for all data elements with a key within given boundaries, commonly denoted as range queries, can be answered. However, to limit sorting to only one dimension can be too restrictive in various applications and hence several approaches [3] including kd-trees and range trees have been developed to overcome those difficulties. In `CHAINER` [4], k-dimensional binary search trees (kd-trees) have been implemented that require $\mathcal{O}(\log(n) + z)$ and $\mathcal{O}(\sqrt{n} + z)$ in time for one- and two-dimensional range queries, respectively, where z elements are within the given range [5]. In contrast, we incorporated the more time-efficient range tree padded with Johnson priority queues.

A range tree is a d-dimensional binary search tree that stores points of the d-dimensional Euclidean space under the condition that there are no two points with an equal coordinate in any dimension [6, pp. 105-116]. A d-dimensional range tree over a set of d-dimensional points consists of a binary search tree using the first coordinate of each point. Moreover, each node v stores an associate structure over the canonical subset $CS(v)$ with each point restricted to its last $d-1$ coordinates. The canonical subset $CS(v)$ of any node v in T consists of all points stored in the leaves of the subtree rooted by v. Finally, an associate structure over $(d-1)$-dimensional points is again a $(d-1)$-dimensional range tree. In the case of 2-dimensional range trees padded with Johnson priority queues, the stratified tree structure consists of a primary binary search tree sorted by the first coordinate padded with Johnson priority queues as associate structures over the canonical subsets sorted by the second coordinate. To answer two-dimensional range queries using this tree structure, first, all splitting nodes are identified whose canonical subsets contain only leaves with a first coordinate within the given first-dimension range. To get the minimal number of splitting nodes, it is demanded that the father of each splitting node is not a splitting node. The minimal set contains at most $\log(n)$ nodes with n leaves in the tree and can be determined by a single top-down traversal. Second, a range query is executed in the associated Johnson queue of each splitting node. Hence, in comparison to common range trees with a worst-time complexity of $\mathcal{O}(\log^2(n) + z)$ for two-dimensional range queries with z points within the given boundaries, the range trees padded with Johnson queues require only $\mathcal{O}(\log(n) \cdot \log(\log(n)) + z)$ in time.

3 Fragment chaining algorithm

We implemented the fragment chaining algorithm, introduced by Abouelhoda et al. [7, 8], in order to find optimal local chains, i.e., sets of non-overlapping ordered fragments with maximal score. While the case of overlapping fragments is explicitly excluded, gaps between fragments are allowed and may be penalized according to different gap cost models. In general, the score of a chain is the sum of their fragment scores minus the penalties for any gaps between adjacent fragments. In the case of local fragment chaining, the fragment is chained to another fragment or chain only if its score is equal to or higher than the necessary gap costs.

Let $f_{beg}.x$, $f_{end}.x$ denote the start and end position of a fragment f in the database sequence x. The start and end positions in the query y are denoted by $f_{beg}.y$ and $f_{end}.y$, respectively. Let f and f' be two non-overlapping ordered fragments, i.e., assume $f_{end}.x < f'_{beg}.x$ and $f_{end}.y < f'_{beg}.y$. Linear gap costs $g_1(f', f)$ between the fragments f and f' are calculated by:

$$g_1(f', f) = \lambda_{g_1} \cdot \Delta_x(f', f) + \epsilon_{g_1} \cdot \Delta_y(f', f) \tag{1}$$

with $\Delta_x(f', f) = |f'_{beg}.x - f_{end}.x - 1|$, $\Delta_y(f', f) = |f'_{beg}.y - f_{end}.y - 1|$, and weighting parameters $\lambda_{g_1}, \epsilon_{g_1} \geq 0$. Hence, for $\lambda_{g_1}, \epsilon_{g_1} > 0$ linear gap costs penalize any distance between fragments on query and database sequence. This scoring system may not be suitable, however, when scattered blocks of local sequence conservation are expected.

The more flexible sum-of-pair gap cost model introduced by Myers and Miller [9] allows to penalize differences of the distances between adjacent fragments on query and database only. The sum-of-pair gap costs $g_{sop}(f', f)$ between non-overlapping ordered fragments f and f' is given by

$$\begin{aligned} g_{sop}(f', f) = \lambda_{g_{sop}} \cdot (&\max\{\Delta_x(f', f), \Delta_y(f', f)\} \\ &- \min\{\Delta_x(f', f), \Delta_y(f', f)\}) \\ &+ \epsilon_{g_{sop}} \cdot \min\{\Delta_x(f', f), \Delta_y(f', f)\} \end{aligned} \tag{2}$$

with parameters $\lambda_{g_{sop}}, \epsilon_{g_{sop}} \geq 0$. Intuitively, $\lambda_{g_{sop}}$ expresses the penalty to align an anonymous character with a gap position while $\epsilon_{g_{sop}}$ is the penalty to align two anonymous characters. With $\epsilon_{g_{sop}} = 0$, the chaining only minimizes the distance difference between fragments.

The chaining algorithm falls into the category of sparse dynamic programming, introduced by Eppstein et al. [10]. Sparse dynamic programming is a technique to design an efficient dynamic programming algorithm in which only a small set of entries within the dynamic programming matrix matters for the optimization of the objective function. Hence, we can take advantage of the sparsity of the fragment space and traverse the list of start and end points sorted by their database position, known as line-sweep. Note that end points are sorted before start points. In consequence, if a start point of a fragment is scanned, the end points of all non-overlapping preceding fragments will already

have been processed. The line-sweep method is used to construct optimal chains in the following way. For any start point, the optimal predecessor to the corresponding fragment is identified by means of range maximum queries (RMQs) over the set of active chains, i.e., set of chains constructed from fragments with already processed end points. For any end point, on the other hand, a novel chain is constructed by connecting the corresponding fragment to its optimal predecessor and marking the end point as active. Note that the optimal predecessor might be non-existent due to the local chaining condition and hence the novel chain consists only of the current fragment. To identify optimal predecessors efficiently, the algorithm and its underlying data structure crucially depends on the selected gap cost model, e.g., linear or sum-of-pair gap costs. Otherwise, the score to each possible predecessor would need to be computed explicitly, yielding a quadratic algorithm. Thus, it is necessary to express the gap costs implicitly in terms of a weight for fragments, denoted as geometric cost of a fragment. They are defined by use of a fixed terminus point t with $t.x \geq \tilde{f}_{end}.x$ and $t.y \geq \tilde{f}_{end}.y$ for any fragment $\tilde{f}$. In the following, the algorithms in both gap cost models vary considerably and hence will be described separately.

Using the linear gap cost model, the geometric cost gc_{g_1} can be assigned to a fragment f in terms of its linear gap costs to the terminus $gc_{g_1} = g_1(t, f_{end})$. In such a way, the predecessor with maximal fragment priority, i.e., the fragments score reduced by its geometric cost, is optimal. During the line-sweep, a Johnson priority queue stores active end points according to their query position y. Note that one-dimensional range queries on the positions in the query sequence are sufficient since only end points with smaller database position can already be active. In addition, an invariant in the Johnson priority queue is retained that end points are only inserted if their predecessor in the queue has a lower fragment priority and successors of inserted end points are deleted if they have a lower fragment priority. Due to this invariant, the identification of the optimal predecessor of an end point requires only one predecessor operation in the Johnson priority queue using its query position.

The implementation of sum-of-pair gap costs, however, is more complicated. This is owed to the distinction of cases in the definition of sum-of-pair gap costs that geometrically corresponds to the first and second octant in the fragment space. Since range queries can only be applied for orthogonal regions, two octant-to-quadrant transformations are introduced and range queries are applied on the set of transformed end points. However, these transformations necessitate two-dimensional rather than one-dimensional range queries that are executed in two range trees padded with Johnson priority queues respectively for both octants. As before, it is possible to assign geometric costs $gc_{g_{sop_1}}$ and $gc_{g_{sop_2}}$ in each octant. The fragment with the maximal fragment priority is then the optimal predecessor in the octant. By retaining an equal invariant in each of the range trees, the identification of the optimal predecessor requires only a top-down traversal to locate splitting nodes in the primary search tree and one predecessor operation in each Johnson queue associated to these nodes. Overall, the fragment chaining algorithm has a worst-time complexity of $\mathcal{O}(n \log(n))$

with linear gap costs and $\mathcal{O}(n \log(n) \log(\log(n)))$ with sum-of-pair gap costs.

Because the database is typically much larger than the query sequence, we introduced a novel clustering approach to facilitate local fragment chaining. According to the definition of local fragment chaining, the fragment score must exceed the necessary gap costs to another fragment or chain to be interlinked. Assuming a positive parameter λ in the linear and sum-of-pair model, an upper limit of the possible database distance between adjacent fragments can be estimated at which the lower bound of their gap costs exceeds the highest possible chain score. Fragments before and after such gap are split up into different clusters where fragments from different clusters may never be part of the same chain. The highest possible chain score max_{score} is estimated during the line-sweep as a product of the maximal observed fragment score per position and an estimate of the query length max_y. Let f and f' be two arbitrary fragments separated by a genomic distance of at least gap, the linear gap costs can be bounded from below by

$$g_1(f', f) = \lambda_{g_1} \cdot \Delta_x(f', f) + \epsilon_{g_1} \cdot \Delta_y(f', f) \geq \lambda_{g_1} \cdot gap. \tag{3}$$

With $\Delta_x(f', f) \geq \Delta_y(f', f)$, the sum-of-pair gap costs between f and f' are lower-bounded as follows.

$$\begin{aligned} g_{sop}(f', f) &= \lambda_{g_{sop}} \cdot (\Delta_x(f', f) - \Delta_y(f', f)) + \epsilon_{g_{sop}} \cdot \Delta_y(f', f) \\ &= \lambda_{g_{sop}} \cdot \Delta_x(f', f) + (\epsilon_{g_{sop}} - \lambda_{g_{sop}}) \cdot \Delta_y(f', f) \\ &\geq \lambda_{g_{sop}} \cdot gap + (\epsilon_{g_{sop}} - \lambda_{g_{sop}}) \cdot \Delta_y(f', f) \\ &\geq \lambda_{g_{sop}} \cdot gap + \min\{0, \epsilon_{g_{sop}} - \lambda_{g_{sop}}\} \cdot max_y \end{aligned} \tag{4}$$

The distance between adjacent fragments is calculated during the line-sweep and if the resulting minimal gap penalty is larger than the current estimate of the highest possible chain score max_{score}, a new cluster is started with the fragment after the gap. In the end, each of the clusters is chained separately, improving both running time and memory consumption. In the worst case, all fragments are in the same cluster leading to the same performance as without clustering.

References

[1] Peter van Emde Boas, R. Kaas, and E. Zijlstra. Design and implementation of an efficient priority queue. *Mathematical Systems Theory*, 10:99–127, 1977.

[2] Donald B. Johnson. A priority queue in which initialization and queue operations take O(log log D) time. *Mathematical Systems Theory*, 15(4):295–309, 1982.

[3] Jon Louis Bentley and Jerome H. Friedman. Data structures for range searching. *ACM Comput. Surv.*, 11(4):397–409, 1979.

[4] M I Abouelhoda and E Ohlebusch. Chainer: Software for comparing genomes. In *Proceedings of the 12th International Conference on Intelligent Systems for Molecular Biology + 3rd European Conference on Computational Biology*, 2004.

[5] Jon Louis Bentley. K-d trees for semidynamic point sets. In *SCG '90: Proceedings of the sixth annual symposium on Computational geometry*, pages 187–197, New York, NY, USA, 1990. ACM.

[6] Mark de Berg, Marc van Kreveld, Mark Overmars, and Otfried Schwarzkopf. *Computational Geometry*. Springer, February 2000.

[7] Mohamed Ibrahim Abouelhoda and Enno Ohlebusch. Multiple genome alignment: Chaining algorithms revisited. In *Combinatorial Pattern Matching: 14th Annual Symposium, CPM 2003, Morelia, Michoacn, Mexico, June 25-27, 2003. Proceedings*, volume 2676/2003 of *Lecture Notes in Computer Science*. Springer Berlin / Heidelberg, 2003.

[8] Mohamed Ibrahim Abouelhoda and Enno Ohlebusch. Chaining algorithms for multiple genome comparison. *Journal of Discrete Algorithms*, 3(2-4):321 – 341, 2005.

[9] Gene Myers and Webb Miller. Chaining multiple-alignment fragments in sub-quadratic time. In *SODA '95: Proceedings of the sixth annual ACM-SIAM symposium on Discrete algorithms*, pages 38–47, Philadelphia, PA, USA, 1995. Society for Industrial and Applied Mathematics.

[10] David Eppstein, Zvi Galil, Raffaele Giancarlo, and Giuseppe F. Italiano. Sparse dynamic programming I: linear cost functions. *J. ACM*, 39(3):519–545, 1992.
